# EMS Interventions during Planned Out-of-Hospital Births with a Midwife: A Retrospective Analysis over Four Years in the Polish Population

**DOI:** 10.3390/jcm12247719

**Published:** 2023-12-15

**Authors:** Mateusz Strózik, Hanna Wiciak, Lukasz Szarpak, Pawel Wroblewski, Jacek Smereka

**Affiliations:** 1 Department of Emergency Medical Service, Wroclaw Medical University, 50-367 Wroclaw, Poland; m.strozik@umw.edu.pl (M.S.); pawel.wroblewski@umw.edu.pl (P.W.); 2 2nd Department and Clinic of Gynaecology and Obstetrics, Wroclaw Medical University, 50-556 Wroclaw, Poland; 3 1st Department and Clinic of Gynaecology and Obstetrics, Wroclaw Medical University, 50-556 Wroclaw, Poland; 4 Henry JN Taub Department of Emergency Medicine, Baylor College of Medicine, Houston, TX 77030, USA; 5 Department of Clinical Research and Development, LUXMED Group, 02-676 Warsaw, Poland

**Keywords:** planned out-of-hospital births, EMS interventions, home births

## Abstract

Planned out-of-hospital births, facilitated by highly skilled and experienced midwives, offer expectant parents a distinct opportunity to partake in a personalized, intimate, and empowering birth experience. Many parents opt for the care provided by midwives who specialize in supporting home births. This retrospective study is based on 41,335 EMS emergency calls to women in advanced pregnancy, of which 209 concerned home birth situations documenting obstetrical emergencies over four years (January 2018 to December 2022), of which 60 involved the assistance of a midwife. Data were obtained from the Polish Central System for Emergency Medical Services Missions Monitoring, encompassing all EMS interventions in pregnant women. The most frequent reason for emergency calls for obstetrical emergencies with the assistance of a midwife was a failure to separate the placenta or incomplete afterbirth (18 cases; 30%), followed by perinatal haemorrhage (12 cases; 20%) and deterioration of the newborn’s condition (8 cases; 13%). Paramedic-staffed EMS teams conducted most interventions (43 cases; 72%), with only 17 (28%) involving the presence of a physician. Paramedics with extensive medical training and the ability to provide emergency care are in a unique position that allows them to play a pivotal role in supporting planned out-of-hospital births. The analysed data from 2018–2022 show that EMS deliveries in Poland are infrequent and typically uncomplicated. Continuing education, training, and adequate funding are required to ensure the EMS is ready to provide the best care. EMS medical records forms should be adapted to the specific aspects of care for pregnant patients and newborns.

## 1. Introduction

Home births have historically been the basis for the survival of our civilization, but the institutionalization of care for the pregnant woman and the newborn, and the desire to provide optimal medical care, has led to the fact that home births in Western civilizations make up a relatively small percentage compared to births taking place in hospitals or other healthcare units. While this traditional approach has deep roots in human history, it prompts contemplation about its safety and efficacy compared to modern medical practices [1,2]. Exploring the historical context reveals a shift from home births to the emergence of hospital deliveries during the 18th and 19th centuries. However, the dynamics of childbirth underwent a paradigm shift with revolutionary insights into bacterial infections, sepsis, and the advent of antiseptic techniques in the mid-19th century. Proponents of home births emphasize the progressive and unnecessary medicalization of perinatal care and the unique experience of home birth, including psychological aspects. At the same time, opponents stress the risks associated with complications of childbirth, as well as risks to mother and child, stressing that these risks can be minimized by planning birth immediately in a hospital or other medical institutions for this purpose. Advocates of home births stress the need for proper qualification for home births to reduce the risk of complications in the case of obstetric abnormalities that are foreseeable [1].

The rate of home births varies significantly across countries. In the Netherlands in 2015 and 2019, the percentage of home births was 16.3%, with the percentage in other countries as follows: 1.4% in Denmark, 1.3% in Germany, 1.1% in Belgium, 0.9% in Hungary, 0.32% in Spain, 0.2% in Finland, and 0.03% in Poland. The Netherlands has the highest rate of home births, which is most likely due to the uniqueness of prenatal care. The healthcare model for pregnant women is bifurcated into primary and secondary care. Patients with a low risk of complications are categorized under primary care, while those with a potential for complications receive specialized care, referred to as secondary care [1]. However, a noticeable trend can be observed in the United States, where planned home births have increased. From 2004 to 2017, there was a 75% surge in the incidence of planned home births [2].

Home births take place in Poland relatively infrequently, and a midwife provides care for a patient who decides to have a home birth. In some cases, pregnant patients decide to deliver without the presence of a medical professional. In almost all cases, births in Poland occur in a hospital setting, and maternity care is free of charge. The problem facing hospitals and maternity wards is the reduction in the number of births, which leads, for economic and organizational reasons, to a reduction in the number of hospitals with obstetric units, which may, in some cases, lead to longer EMS transport times for a patient during delivery to the nearest hospital with an obstetric unit.

The system of emergency medical services in Poland is based on paramedic emergency medical teams, which include paramedics trained by legal requirements; an increasing number of paramedics in Poland have a bachelor’s degree in medical rescue, and during their studies, there are courses in obstetrics and the practical and theoretical aspects of providing emergency care to women in pregnancy-related emergencies, as well as delivering a baby in a prehospital setting. An decreasing number of teams include physicians, a minority of whom specialize in emergency medicine. The Helicopter Emergency Medical Service system is based on the presence of a physician specializing in or having specialized in emergency medicine and a paramedic with very high professional qualifications.

For EMS personnel, the primary focus should be promptly transporting the mother to a hospital that possibly offers tertiary care of obstetrics while providing the necessary emergency actions, including medical care for the pregnant woman, the ability to deliver the baby, and care for the mother and the newborn. In such controlled settings, specialized healthcare providers can deliver the baby, ensuring the availability of appropriate equipment and expertise to manage any potential complications. In many studies, it is emphasized that unplanned birth outside a medical facility is linked to a higher peripartum mortality rate. Emergency medical teams also handle cases such as eclampsia/HELLP, which pose a direct threat to the life of both the mother and the child [3,4].

It is worth noting that up to one-third of nulliparous patients may require transfer to a hospital setting. The chance that multiparous females will have to be transferred to the hospital is significantly lower at about 8% [5]. Therefore, birth plans should include contingencies for urgent and nonurgent transport to a nearby hospital, which, through proper organizational measures, will reduce the time to reach the hospital and, thus, the risk in the event of the transfer to the hospital [6].

However, in some cases, there may be insufficient time to transport the mother to an appropriate medical facility. Unplanned prehospital deliveries have been associated with increased perinatal mortality and morbidity, posing risks to both the newborn and the mother. It should be noted that in the case of unplanned births, the risk of death of the newborn in the first week of its life may be even five times higher [7].

Whether homebirth is beneficial remains a topic of ongoing debate. A consensus is needed among scientific organizations, with contradictory recommendations provided. When assessing the feasibility of home births, many factors should be taken into account, including the course of the pregnancy and obstetric history, the ability to provide professional midwifery care at home, the potential time to reach the hospital, and most importantly, the likelihood of a smooth, natural home birth taking into account the wishes of the pregnant woman and her awareness of the risks and benefits [8,9]. 

In planned out-of-hospital births, the need for transfer to hospital care may arise during labour due to cord prolapse, haemorrhage, malpresentation, maternal exhaustion, slow labour progress, foetal distress, and suspected intra-amniotic infection. In some cases, the following may occur after childbirth: low Apgar score, congenital malformation, suspected sepsis, respiratory distress, lacerations requiring repair by an obstetrician, or retained placenta [9,10]. Other authors explicitly highlight the correlation between unplanned births outside a medical facility and an elevated peripartum mortality rate [11].

Emergency medical service (EMS) providers are called upon to deliver prompt medical attention and facilitate transportation in birth complications requiring emergency care for a mother or a child. However, many paramedics note issues that affect them, such as a lack of adequate training, a mismatch between societal expectations regarding EMTs and the reality they encounter in out-of-hospital maternity care, difficulties in deciding whether to transport a patient or manage childbirth on-site in challenging situations, and even a lack of understanding from midwives when transferring a patient to the hospital. All these factors contribute to exceptional challenges and stress in providing care [12].

This study aimed to assess the rate of EMS interventions in planned home births in Poland and analyse the characteristics of patients who require EMS assistance and the scope of response by EMS teams for complicated planned home births.

## 2. Materials and Methods

We conducted a cross-sectional study based on the National Centre for Emergency Medical Services data from 2018–2022 encompassing all interventions conducted by EMS within the country. This register held by the National Centre for Emergency Medical Services covers all rescue actions undertaken by Emergency Medical Services teams in Poland in a unified manner according to a set form; the introduction of this system made it possible to make analyses and data comparisons. The period of analysis we performed covered the years in which all EMS team rescue actions were subject to the same record-keeping rules.

Inclusion criteria in the study were emergency team calls to the patient in planned home births with a midwife, regardless of the reason for the call. The analysis did not consider exclusion criteria; the only inclusion criteria were planned home births with a midwife. 

Due to no diagnostic code reliably identifying OOH (out-of-hospital) delivery, we utilized multiple search strategies to identify planned out-of-hospital births. Two independent researchers manually conducted searches for information describing planned OOH births with a midwife.

The original database was in the range of ICD-10 codes between O30–O92 to isolate cases of pregnant women. This range of codes allowed the inclusion of maternal care related to the foetus and amniotic cavity and possible delivery problems, complications of labour and delivery, and delivery complications predominantly related to the puerperium.

Subsequently, the database underwent manual searches conducted by two independent researchers to identify patients who had planned home deliveries. Home births were identified by manually analysing the selected records, their contents, descriptions, and EMS rescue actions taken. Within the category of home births, a specific subgroup was distinguished consisting of cases where there was documented information regarding a planned birth involving the participation of a midwife.

Before conducting the research, the study was approved by the Bioethics Committee of the Wroclaw Medical University, Poland (Approval No: KB-975/2022). The analysed database did not contain information that would enable the identification of the patient and the emergency medical team providing care to the patient.

## 3. Results

Between 2018 and 2022, 41,335 EMS interventions involving pregnant patients were recorded using ICD-10 codes within the range of O30–O92. Of the identified interventions associated with planned childbirth, 209 cases were documented in EMS medical records at the scene and during the transfer to the hospital. Among these cases, only 60 involved the presence and assistance of a midwife, and further analysis is focused on this group of patients.

**Figure 1:**
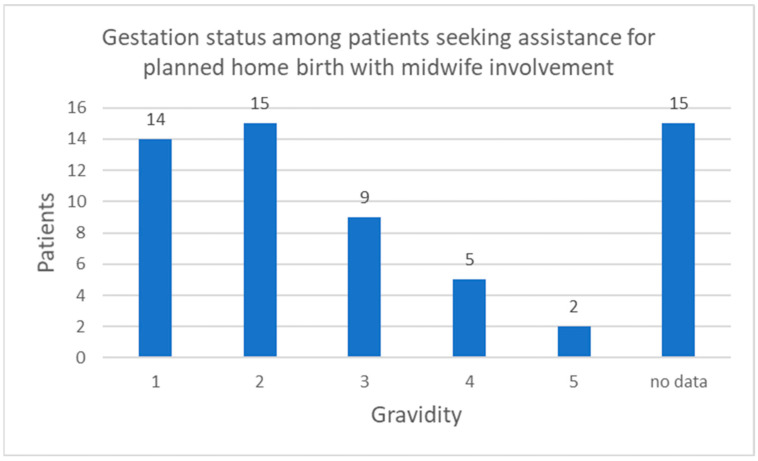
Gravidity of patients calling for help with a planned home birth with the participation of a midwife.

The age range of the patients varied from 23 to 44 years. On average, the patient’s age was 33 years. The description of the EMS mission lacked information on gravidity in 15 cases (25%). Of the patients, 31 were multiparous, including 15 who had experienced two pregnancies. Additionally, there were 14 visits recorded for primiparous patients. Detailed information on the gravidity of patients calling for help is shown in Figure 1.

Table 1 shows the reasons for EMS calls to patients who had a planned delivery with midwife care. Retained placenta (30.0%) and postpartum haemorrhage (21.7%) were the most common reasons for EMS team calls, followed by newborn’s condition deterioration (13.3%), mothers’ birth injuries (11.7%), abnormal delivery mechanism (5.0%), abnormal foetal heart rate (5.0%), breech delivery (3.3%), and shoulder dystocia (1.7%), with the reason for the call not documented in 8.3% of cases. 

**Figure 2:**
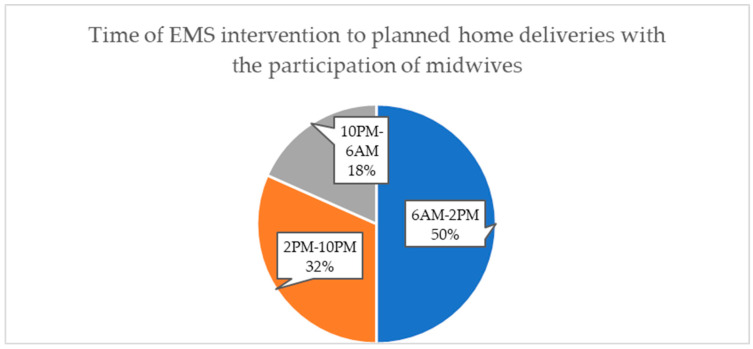
The number of interventions for planned home deliveries with the participation of midwives based on different time intervals.

We categorized the time of interventions into three different time frames: between 6 AM and 2 PM; between 2 PM and 10 PM; and at night, between 10 PM and 6 AM. Half of the interventions occurred between 6 AM and 2 PM. Figure 2 contains detailed data.

The majority of interventions occurred with the involvement of the core team, comprising a minimum of two medical professionals authorized to perform medical actions, including a system nurse or a paramedic, accounting for 43 cases (72%). In contrast, teams including a physician comprised only 17 cases (28%).

Out of the total 60 interventions, in 7 cases, the delivery was not advanced; therefore, the EMS team managed to transport the patient to the hospital. In the remaining 53 interventions, the delivery occurred either before the EMS teams arrived or during their presence.

**Figure 3:**
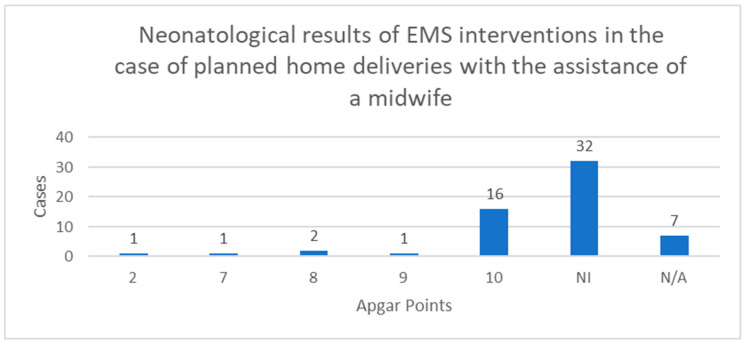
The neonatological outcomes of EMS interventions in planned home deliveries with the assistance of a midwife. NI—No information. N/A—Not applicable.

Figure 3 shows neonatological outcomes of EMS interventions in planned home births with the assistance of a midwife. Apgar scores were not provided in most of the reports submitted by EMS teams. Among the cases where Apgar scores were assessed, 16 received 10 points, one scored 9 points, two were scored at 8 points, one received 7 points, and one was at 2 points. 

## 4. Discussion

Our findings highlight the crucial role of emergency medical teams in prehospital care for pregnant women, analysing the basic medical challenges and looking at the role of teams with no physicians. In our study, the most frequent reason for emergency calls for obstetrical emergencies with the assistance of a midwife was a failure to separate the placenta or incomplete afterbirth, followed by perinatal haemorrhage and deterioration of the newborn’s condition. Paramedic-staffed EMS teams conducted most interventions, with only 28% involving the presence of a physician. Half of the interventions took place between 6 AM and 2 PM. 

In the studies conducted by Eisenbrey et al., the primary reasons for EMS team intervention were as follows: postpartum haemorrhage (8 cases, 25.0%), non-transient apnoea including CPR (4 cases, 12.5%), foetal demise (2 cases, 6.3%), abnormal presentation of congenital anomaly (2 cases, 6.3%), and others. However, our research does not specifically reference these findings. In our analysis, we included home births attended by midwives, but in the study by Eisenbrey et al., only 31 out of 223 births in the presence of EMS teams were attended by a midwife. In this study, in the case of EMS teams being called for home births in the presence of a midwife, the study showed a high rate of complications (61.3%), which is undoubtedly related to the fact that no EMS team was called for an adequately precipitated home birth attended by a midwife. The most common reasons for intervention in our study were retained placenta (18 cases, 30%), postpartum haemorrhage (13 cases, 22%), and newborn condition deterioration (8 cases, 13%). Nonetheless, it is undeniable that each of the above situations was a significant challenge and stressful for EMS teams [13].

Bernhardt et al., in their research, reported 40 interventions over five years. The results of the study in Germany are similar to those we assessed. However, it is essential to note a significant disparity regarding the timing of these interventions. Seventy-three percent of the interventions occurred during nighttime hours between 4 PM and 7 AM. In contrast, our data indicate that 50% of the interventions occurred between 6 AM and 2 PM [14]. The emergency medical system in Poland is generally based on 12-h shifts from 7 AM to 7 PM and 7 PM to 7 AM. However, our proposed time slots facilitate better identification of deliveries and interventions at night and indicate that home births are more likely to occur during daytime hours, which are the typical daytime working hours of the medical personnel. Several studies have shown that interventions by EMS teams to patients delivering in out-of-hospital settings mainly occur at night. However, it should be noted that in our study, we only analysed home births, where the final decision by the parturient regarding the place of delivery (home or hospital) may have depended on the hour at which labour began or the anticipated end of labour, as well as the availability of medical personnel who could attend the home birth, including primarily the midwife.

Women often place their trust in the natural process of birth [15]. Furthermore, motivations for opting for home births may stem from the belief that hospital personnel are unsupportive of natural physiological birth processes, rely heavily on unnecessary interventions, and may not prioritize patient preferences [16,17]. 

The decline in maternal mortality rates has created a perception that pregnancy is now entirely safe, causing some to overlook the advancements in hospital care that have contributed to this improvement. However, it is essential to recognize that our ability to save lives in medical facilities has significantly evolved. Despite this progress, a growing movement advocates for home births, often defined as one of women’s rights, which is noticeable in the research of Catlings et al. [18]. Proper selection of patients delivering at home, providing opportunities for support by professional staff in home conditions, and considering transportation options are primary factors affecting safety.

Women chose OOH birth primarily due to a sense of enhanced safety, the desire to avoid unnecessary medical interventions often associated with hospital births, previous negative experiences in hospitals, the need for personal control, and the comfort and familiarity of their environment [15]. A suggested option to consider is to undertake such organizational and facility measures so that a woman giving birth in a hospital is provided with professional care while reducing the stress of being in a hospital environment. The right approach of the personnel and the atmosphere and care, including the opinions of other parturients about the unit, can influence patients’ decisions about where to give birth.

In the Netherlands, scientific studies have indicated that planned home births exhibit comparable levels of safety to planned hospital births [15]. This is also confirmed by other studies in different countries, which did not show an increase in neonatal morbidity and mortality associated with planned home births [19,20,21]. This can result from proper obstetric qualification and perinatal care, which can ensure that patients with an increased risk of perinatal complications do not undertake planned home birth. Studies indicating that there is no difference in obstetric complications and the newborn’s condition between home and hospital births are based mainly on the good qualification of patients and the management of pregnancy, which ensures that potentially complicated births will take place in a hospital setting.

Additionally, maternal outcomes, in the case of planned home births, are characterized by reduced interventions and complications [22,23]. However, due to the relatively small percentage of home births, it is difficult to draw definitive conclusions based on these studies, as severe complications were very rare in home births and in hospital settings.

Several studies, including the work of G. McLelland et al., highlight that most interventions conducted by paramedics during childbirth involve uncomplicated cases. It is crucial to acknowledge that, apart from spontaneous deliveries that necessitate no intervention, there are unique circumstances where specialized care is essential to prevent potential risks to the health or life of both the mother and the newborn [24]. This study showed that over 88% of out-of-hospital births were uncomplicated deliveries at term. This analysis showed that the most common perinatal problems were postpartum haemorrhage, breech, cord prolapse, prematurity, and neonatal death. Interestingly, in more than 16% of the cases, the labouring women had a complicated medical history that could affect the delivery. Most deliveries took place at night between 10 PM and 6 AM.

The most common procedures that emergency medical teams perform during intervention cases of pregnant women are pulse oximetry, medical history, and blood pressure measurement [25]. These are the typical actions taken to assess vital functions in any patient in an EMS operation.

In a large study that analysed data from Finland from 1996 to 2013, the authors identified an issue similar to the one observed in our study. The lack of standardized documentation for out-of-hospital deliveries and the flexibility in completing the records resulted in specific gaps within the documentation process. Consequently, the analysis of Apgar scores could not be conducted in their study due to missing data. This problem was solved by creating a birth register in 2004, in which this information must be included [26]. This study and the measures taken point to similar problems with the form of documentation and the importance of the correct assessment of the newborn’s condition and data entry.

Our preliminary observations indicate that midwife-attended births can pose unique challenges for emergency medical services (EMS). Although EMS team personnel are prepared to deliver normal births and emergencies in the case of complications, the limited options for taking action, including action related to obstetric emergencies, can be a significant challenge for staff. In Poland, paramedic teams are not equipped as standard with oxytocin or other drugs with similar effects, nor is it a drug that can be administered by a paramedic alone without a physician’s consent.

Due to the relatively infrequent nature of responding to women in labour compared to other EMS interventions, the teams may need more readiness to handle obstetric complications, as indicated by incompletely filled-out patient charts. Paramedics face numerous occupational hazards daily, with the most significant ones in terms of serious disease development being exposure to harmful biological factors, musculoskeletal risk factors, fatigue, and mental overload associated with their occupational responsibilities. Despite shouldering such burdens, transporting a labouring woman or a distressed baby poses one of the most challenging situations for healthcare professionals, particularly those without midwifery experience [27,28]. This is due to both legal responsibility and societal expectations, which sometimes do not take into account the natural occurrence of severe obstetric complications with high mortality rates, largely regardless of where medical care is provided. In the setting of prehospital care, these opportunities are particularly limited compared to the conditions of a well-equipped and well-staffed multi-profile hospital.

In our study, we did not observe any patient deaths; however, we encountered a case in which a patient’s delivery was managed by a midwife, resulting in the child being born in a highly critical condition. A Norwegian study detected that a significant percentage of the liveborn cases died due to abuse, i.e., in indistinct circumstances [29]. Such cases appear challenging to prevent and are unlikely to be significantly influenced by the proximity to the nearest delivery unit. Mental health issues, substance abuse, and social exclusion are potential contributing factors in these instances. To improve pregnancy outcomes in these complex situations, comprehensive multidisciplinary interventions are necessary for the mother and her family.

As for future research directions and possible applications of the research, in our opinion, it is necessary to include the specifics of care for the pregnant patient and the newborn in the documentation of EMS teams, which should enhance the quality of documentation and the quality of care. A detailed documentation scheme covering obstetric emergencies facilitates both the maintenance of medical records and makes it easier for paramedics to take a more extensive medical history related to the current pregnancy and previous pregnancies. It can facilitate a better assessment of the condition of the mother, foetus, or newborn, as well as specific risks in pregnancy. Further analysis will be needed to determine whether expanding EMS team forms including specific forms regarding the pregnant patient, delivery, and newborn care helps facilitate care delivery and documentation analysis. 

## 5. Limitations

This study has some limitations primarily attributed to its data source. The data from narrative summaries written by EMS providers at the scene and during the transfer to the hospital and collected in the database are restricted due to a specific structure of prehospital emergency medical charts, which is not specifically adjusted to the state of pregnancy and labour complications. Therefore, these circumstances make EMS teams improvise, resulting in varying data quality concerning resuscitative efforts or prenatal care. The dataset was limited in its ability to reliably identify the mother who had just delivered and the infant, as some records combined documentation for both patients. In contrast, others documented separate records for each case. Not all intervention descriptions contained sufficient data, including important information such as the Apgar score of newborns or data directly associated with pregnancy. This limitation arises from using a universal form by EMS that was not specifically tailored for pregnant patients. In order to ensure the comprehensive capture of important interview information during interventions with pregnant women, it appears logical to establish a distinct card specifically designed to document these events. Implementing such a measure can enhance the accuracy and completeness of data related to visits involving pregnant patients.

## 6. Conclusions

The analysed data from 2018–2022 shows that EMS deliveries in Poland are infrequent and typically uncomplicated. However, if complications occur, they often put paramedics in a challenging situation. Therefore, continuing education, training and adequate funding are required to ensure the EMS is ready to provide the best possible care. EMS medical records forms should be adapted to the specific aspects of care for pregnant patients and newborns.

## Figures and Tables

**Table 1:** Reasons for calling EMS for planned home births conducted by midwives.

Complication	N	%
Retained placenta	18	30.0
Postpartum haemorrhage	13	21.7
Newborn’s condition deterioration	8	13.3
Mothers’ birth injuries	7	11.7
N/A	5	8.3
Abnormal delivery mechanism	3	5.0
Abnormal foetal heart rate	3	5.0
Breech delivery	2	3.3
Shoulder dystocia	1	1.7

## Data Availability

The Polish Ministry of Health holds administrative control and authority over the data obtained from the Central System for EMS Missions Monitoring, forming this study’s foundation. The Ministry provided the clinical data for this study after an individual institutional request to access the database.

## References

[1] Galková G., Böhm P., Hon Z., Heřman T., Doubrava R., Navrátil L. (2022). Comparison of Frequency of Home Births in the Member States of the EU Between 2015 and 2019. Glob. Pediatr. Health.

[2] MacDorman M.F., Declercq E. (2019). Trends and state variations in out-of-hospital births in the United States, 2004–2017. Birth.

[3] Josephsen J.B., Kemp J., Elbabaa S.K., Al-Hosni M. (2015). Life-Threatening Neonatal Epidural Hematoma Caused by Precipitous Vaginal Delivery. Am. J. Case Rep..

[4] Engjom H.M., Morken N.H., Høydahl E., Norheim O.F., Klungsøyr K. (2018). Risk of eclampsia or HELLP-syndrome by institution availability and place of delivery—A population-based cohort study. Pregnancy Hypertens..

[5] Blix E., Kumle M.H., Ingversen K., Huitfeldt A.S., Hegaard H.K., Ólafsdóttir Ó.Á., Øian P., Lindgren H. (2016). Transfers to hospital in planned home birth in four Nordic countries—A prospective cohort study. Acta Obstet. Gynecol. Scand..

[6] American College of Nurse-Midwives (2016). Midwifery Provision of Home Birth Services: American College of Nurse-Midwives. J. Midwifery Womens. Health.

[7] Gunnarsson B., Smárason A.K., Skogvoll E., Fasting S. (2014). Characteristics and outcome of unplanned out-of-institution births in Norway from 1999 to 2013: A cross-sectional study. Acta Obstet. Gynecol. Scand..

[8] American College of Nurse-Midwives (2016). Position Statement—Planned Home Birth. www.midwife.org.

[9] ACOG Planned Home Birth. https://www.acog.org/clinical/clinical-guidance/committee-opinion/articles/2017/04/planned-home-birth.

[10] Catling C., Dahlen H., Homer C.S.E. (2014). The influences on women who choose publicly-funded home birth in Australia. Midwifery.

[11] Engjom H.M., Morken N.H., Høydahl E., Norheim O.F., Klungsøyr K. (2017). Increased risk of peripartum perinatal mortality in unplanned births outside an institution: A retrospective population-based study. Am. J. Obstet. Gynecol..

[12] Vagle H., Haukeland G.T., Dahl B., Aasheim V., Vik E.S. (2019). Emergency medical technicians’ experiences with unplanned births outside institutions: A qualitative interview study. Nurs. Open.

[13] Eisenbrey D., Dunne R.B., Fales W., Torossian K., Swor R. (2022). Describing Prehospital Deliveries in the State of Michigan. Cureus.

[14] Eisenbrey D., Dunne R.B., Fales W., Torossian K., Swor R. (2009). Prähospitale geburtshilfliche Notfälle in einem bodengebundenen städtischen Notarztsystem: Retrospektive Analyse eines 5-jährigen Zeitraums. Anaesthesist.

[15] Boucher D., Bennett C., McFarlin B., Freeze R. (2009). Staying Home to Give Birth: Why Women in the United States Choose Home Birth. J. Midwifery Womens. Health.

[16] Cheyney M., Bovbjerg M., Everson C., Gordon W., Hannibal D., Vedam S. (2014). Outcomes of care for 16,924 planned home births in the United States: The Midwives Alliance of North America Statistics Project, 2004 to 2009. J. Midwifery Womens. Health.

[17] Zielinski R., Ackerson K., Low L.K. (2015). Planned home birth: Benefits, risks, and opportunities. Int. J. Womens. Health.

[18] Stapleton S.R., Osborne C., Illuzzi J. (2013). Outcomes of care in birth centers: Demonstration of a durable model. J. Midwifery Womens. Health.

[19] Blix E., Huitfeldt A.S., Øian P., Straume B., Kumle M. (2012). Outcomes of planned home births and planned hospital births in low-risk women in Norway between 1990 and 2007: A retrospective cohort study. Sex. Reprod. Healthc..

[20] Cox K.J., Schlegel R., Payne P., Teaf D., Albers L. (2013). Outcomes of planned home births attended by certified nurse-midwives in southeastern Pennsylvania, 1983-2008. J. Midwifery Womens. Health.

[21] De Jonge A., Geerts C.C., Van Der Goes B.Y., Mol B.W., Buitendijk S.E., Nijhuis J.G. (2015). Perinatal mortality and morbidity up to 28 days after birth among 743 070 low-risk planned home and hospital births: A cohort study based on three merged national perinatal databases. BJOG.

[22] Kataoka Y., Eto H., Iida M. (2013). Outcomes of independent midwifery attended births in birth centres and home births: A retrospective cohort study in Japan. Midwifery.

[23] Kennare R.M., Keirse M.J.N.C., Tucker G.R., Chan A.C. (2010). Planned home and hospital births in South Australia, 1991–2006: Differences in outcomes. Med. J. Aust..

[24] McLelland G., McKenna L., Morgans A., Smith K. (2018). Epidemiology of unplanned out-of-hospital births attended by paramedics. BMC Pregnancy Childbirth.

[25] Rzońca E., Bień A., Wejnarski A., Gotlib J., Bączek G., Gałązkowski R., Rzońca P. (2022). Suspected Labour as a Reason for Emergency Medical Services Team Interventions in Poland—A Retrospective Analysis. Healthcare.

[26] Ovaskainen K., Ojala R., Tihtonen K., Gissler M., Luukkaala T., Tammela O. (2020). Unplanned out-of-hospital deliveries in Finland: A national register study on incidence, characteristics and maternal and infant outcomes. Acta Obstet. Gynecol. Scand..

[27] Khazaei A., Esmaeili M., Navab E. (2019). The Most and Least Stressful Prehospital Emergencies from Emergency Medical Technicians’ View Point; a Cross-Sectional Study. Arch. Acad. Emerg. Med..

[28] Gonczaryk A., Chmielewski J.P., Strzelecka A., Fiks J., Witkowski G., Florek-Luszczki M. (2022). Occupational Hazards in the Consciousness of the Paramedic in Emergency Medical Service. Disaster Emerg. Med. J..

[29] Gunnarsson B., Fasting S., Skogvoll E., Smárason A.K., Salvesen K. (2017). Why babies die in unplanned out-of-institution births: An enquiry into perinatal deaths in Norway 1999–2013. Acta Obstet. Gynecol. Scand..

